# Josephson Diode Effect in High-Mobility InSb Nanoflags

**DOI:** 10.1021/acs.nanolett.2c02899

**Published:** 2022-10-26

**Authors:** Bianca Turini, Sedighe Salimian, Matteo Carrega, Andrea Iorio, Elia Strambini, Francesco Giazotto, Valentina Zannier, Lucia Sorba, Stefan Heun

**Affiliations:** † NEST, Istituto Nanoscienze-CNR and Scuola Normale Superiore, 56127Pisa, Italy; ‡ CNR-SPIN, 16146Genova, Italy

**Keywords:** Josephson junctions, Supercurrent Diode Effect, Spin−orbit coupling, InSb, Nb

## Abstract

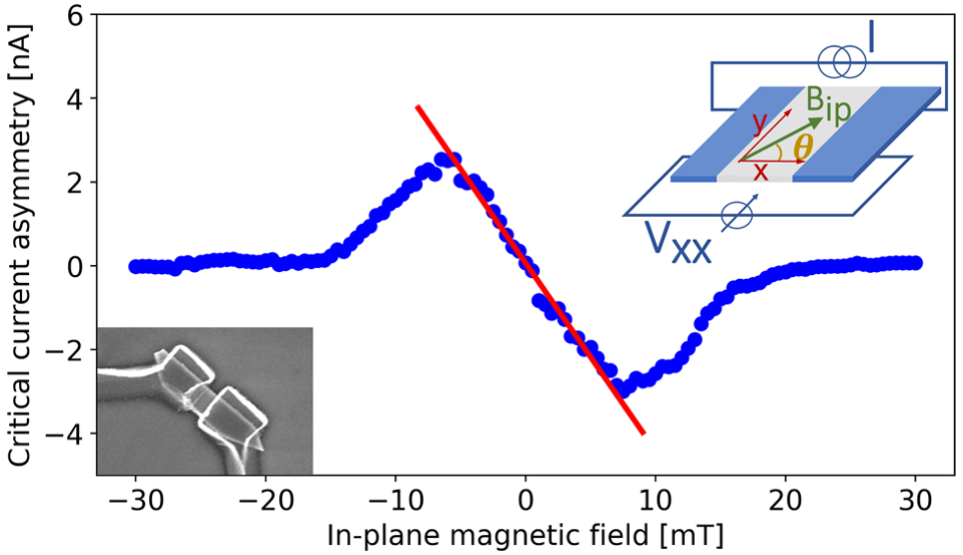

We report nonreciprocal dissipation-less transport in single ballistic InSb nanoflag Josephson junctions. Applying an in-plane magnetic field, we observe an inequality in supercurrent for the two opposite current propagation directions. Thus, these devices can work as Josephson diodes, with dissipation-less current flowing in only one direction. For small fields, the supercurrent asymmetry increases linearly with external field, and then it saturates as the Zeeman energy becomes relevant, before it finally decreases to zero at higher fields. The effect is maximum when the in-plane field is perpendicular to the current vector, which identifies Rashba spin–orbit coupling as the main symmetry-breaking mechanism. While a variation in carrier concentration in these high-quality InSb nanoflags does not significantly influence the supercurrent asymmetry, it is instead strongly suppressed by an increase in temperature. Our experimental findings are consistent with a model for ballistic short junctions and show that the diode effect is intrinsic to this material.

Nonreciprocal charge transport is at the heart of conventional electronics, in which a fundamental building block, the diode, is based on the p–n junction. In such systems, rectification takes place due to the presence of a heterojunction that explicitly breaks inversion symmetry. Only very recently it has been proposed that the superconducting analogue of nonreciprocal transport can be made,^1^ based on similar symmetry arguments: in this case, nonreciprocity is expected when time-reversal and inversion symmetries are simultaneously broken.^2−6^ Supercurrent rectification has been achieved in superconducting quantum interference devices (SQUIDs), where the flux tunability allows high rectification coefficients to be reached. However, this rectification is of extrinsic nature, being induced by asymmetric junctions and the presence of an external flux threading the SQUID.^7−10^

In fact, an intrinsic supercurrent analogue exists—the supercurrent diode effect (SDE)—whose exploitation would constitute a breakthrough for low-temperature technology and superconducting electronics. The first experimental report on the SDE, based on electrically polar materials,^11^ has appeared very recently, demonstrating supercurrent rectification. Soon after, other systems^12−23^ have been inspected looking at supercurrent nonreciprocal transport, complemented by theoretical efforts,^9,24−32^ to shed light on the microscopic mechanisms responsible for the SDE. However, both from an experimental and a theoretical point of view, this field is still in its infancy.

During the past decade, there has been a widespread interest in the physics of hybrid systems comprising superconductors and low-dimensional semiconductors featuring strong spin–orbit coupling (SOC). Indeed, these systems offer an ideal platform to develop new architectures able to coherently control electron spin with significant impact in spintronics and topological quantum computing.^15,33−35^

Exploiting the large SOC of InAs, the authors of ref (13) have observed SDE in an array of Josephson junction (JJ) devices. Supercurrent rectification in hybrid JJs has been also referred to as the Josephson diode effect (JDE). Here, the combination of SOC and superconducting proximity leads to a strong interaction between spin, charge, and superconducting phase, which is the working principle of the φ_0_ junction. In such devices, the current–phase relation (CPR) is shifted by an anomalous phase φ_0_.^36^ Moreover, φ_0_ junctions can be considered the precursors of the Josephson diode: as discussed in ref (13), highly transmissive junctions, which operate in the short-junction regime, are characterized by a skewed CPR, which leads to supercurrent rectification in the presence of an anomalous phase shift.

In this context, InSb represents a valid platform. InSb has a narrow band gap (0.23 eV),^33,37,38^ and a small effective mass (m* = 0.018 *m*_*e*_),^33,39−44^ and exhibits a strong SOC and a large Landé *g*-factor .^45^ In InSb 2D nanostructures, a similar value is measured in the out-of-plane direction, while the in-plane value *g*_ip_^*^ is reduced by a factor of 2, independently of the crystallographic direction .^33,41,44^ Moreover, a Rashba spin–orbit strength of α_R_ ∼ 0.42 eV Å was reported for InSb nanosheets,^34^ which yields a spin–orbit energy  μeV.

In this work, we present the first report of JDE in single planar JJs based on high-quality InSb nanoflags. These structures^46,47^ have been used to form ballistic planar JJs, upon deposition of superconducting contacts.^43,48^ Owing to their intrinsic strong SOC and sizable superconducting proximity,^41,46,47,49,50^ they become a natural platform to investigate JDE and obtain insight on its microscopic mechanism.

Previous experiments on analogous devices show a Nb-induced gap of Δ* = 160 μeV.^48^ This value is close to *E*_so_, suggesting that SOC plays a relevant role in the physics of these InSb JJs. The high quality of the material is a crucial feature that permits it to work in the ballistic regime, allowing for the direct observation of a nonreciprocal supercurrent. In addition, the dependence of the JDE on external parameters can provide valuable information on the symmetry-breaking mechanisms at play. Our observations are consistent with a dominant Rashba coupling related to structural inversion asymmetry. We provide a direct demonstration of JDE in a single and scalable planar JJ, which constitutes a crucial step forward in the understanding of the JDE mechanism.

The system under investigation is a superconducting–normal metal–superconducting (SNS) planar JJ, where the N region consists of an InSb quasi-2D nanostructure. The two devices discussed in this publication (G4 and G5) are operating in the short ballistic regime. Details on device fabrication and characteristics are provided in the Supporting Information (SI). Figure 1b shows a characteristic *V*–*I* curve of device G4. We can clearly distinguish the switching (*I*_sw_) and the retrapping (*I*_rt_) currents.

**Figure 1:**
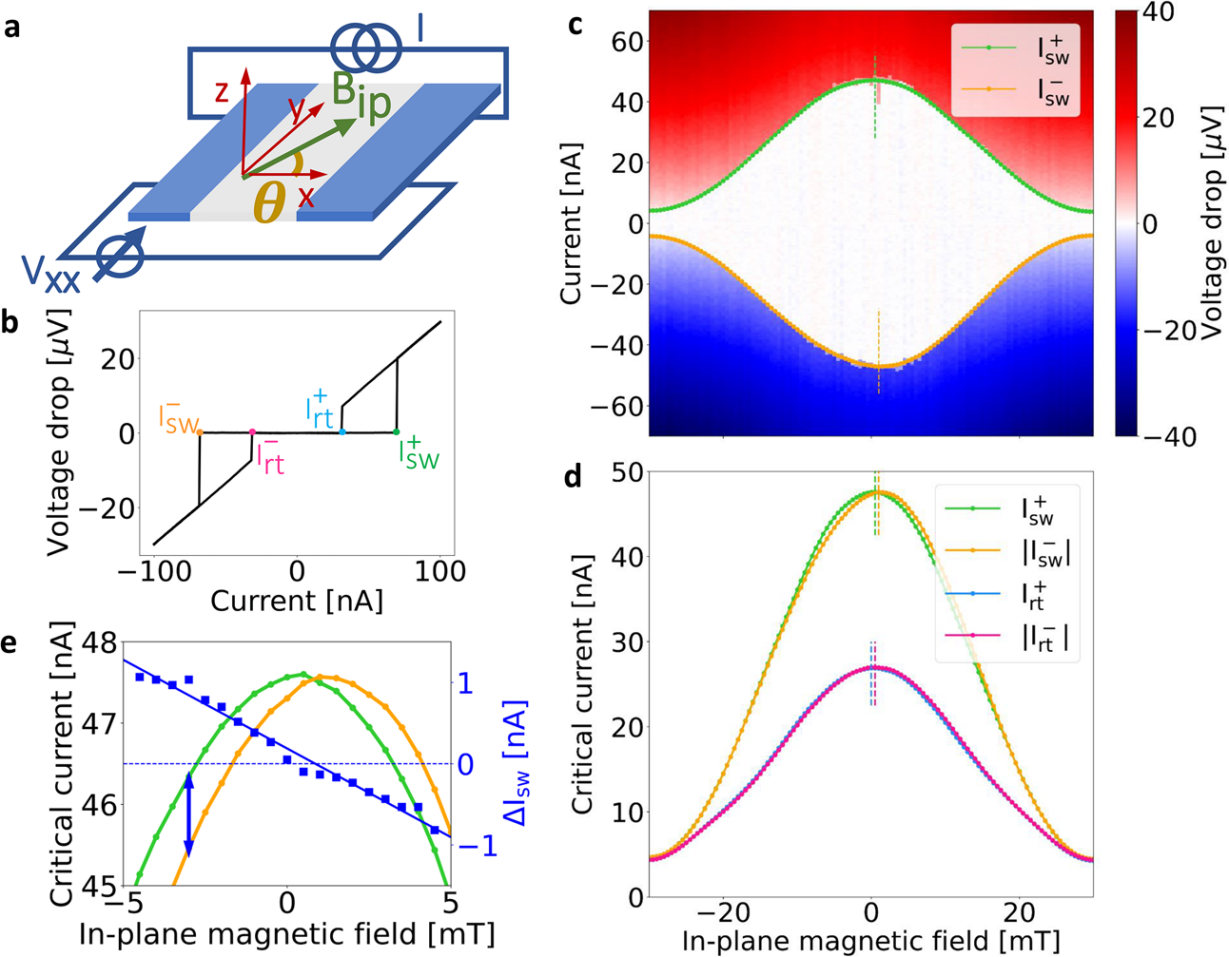
Switching current dependence on in-plane magnetic field. (a) Sketch of the measurement schematics. Also the angle θ between the orientation of the in-plane magnetic field *B*_ip_ and the direction of current flow *I* is indicated. (b) *V*–*I* characteristics at *T* = 30 mK, *B* = 0. The difference between switching and retrapping current, defined in the plot, is clearly visible. (c) Voltage drop across the junction versus applied current bias *I* and in-plane magnetic field *B*_ip_. The green (orange) dots indicate the positive (negative) switching currents, as defined in the main text. (d) The switching current demonstrates a clear asymmetry between the positive and negative branches, shown in green and orange, respectively. The blue and pink lines correspond to the positive and negative retrapping current. In both cases, the maximum of the curves is different: the negative branch is higher for positive values of the magnetic field, and the relation is reversed for negative field. (e) The differences in switching current are better seen in a zoom-in of panel d to the region around *B*_ip_ ∼ 0. The panel also shows that  changes linearly with in-plane magnetic field around *B*_ip_ = 0. The blue arrow visualizes the definition of Δ*I*_sw_. There is a small global field offset (0.75 mT) that we attribute to a residual magnetization of the cryostat. Device G5, angle θ = 129°, *T* = 30 mK, and *V*_bg_ = 40 V.

We report evidence of the JDE in these devices, showing that it only requires an in-plane magnetic field orthogonal to the direction of current flow. Figure 1c shows the voltage drop across junction G5 versus applied current bias *I* and in-plane magnetic field *B*_ip_, with the relative angle set to θ = 129° (cf., Figure 1a). The data was taken by increasing the bias from zero to positive (negative) values, to exclude a current heating of the device before the switching event. The superconducting region, defined by dissipation-less charge transport, corresponds to the white area. The supercurrent is maximum around zero in-plane magnetic field and decreases with increasing field until *B*_ip_ = ± 30 mT, for which it is nearly but not completely suppressed.

From the map, positive and negative switching currents can be extracted. The values of positive switching current *I*_sw_^+^ and negative switching current *I*_sw_^–^ are included in Figure 1c as green and orange dots, respectively. Careful analysis shows that the pattern is slightly skewed with opposite polarity for the two sweep directions. The position of the maximum (minimum) value of *I*_sw_^+^ (*I*_sw_^–^) is indicated in the panel by dashed lines. Note that the two sweep directions are measured consequentially for each value of *B*_ip_; hence, a simple residual magnetization could not explain the opposite skewness of the two patterns. Interestingly, the maximum of the switching current is not observed for zero magnetic field, as one would expect for a standard Fraunhofer-like pattern, but is slightly shifted to a finite magnetic field whose sign depends on the sweeping direction. Therefore, we observe that the magnitude of the positive (negative) switching current *increases* with respect to the value at zero field for small negative (positive) values of the magnetic field.

The asymmetry between the positive and negative branches is more clearly visible by comparing the absolute values of the two curves, shown in Figure 1d,e. For negative magnetic field, , while for positive field, . Thus, for nonzero values of *B*_ip_, there exists a range of bias current values for which the transport across the junction is nondissipative only in one direction, indicating the presence of JDE. In addition, the action of the Josephson rectifier is reversed with the sign of the magnetic field. In the same measurement, also the retrapping current was recorded when sweeping the current back to zero after each switching event. These data are shown in Figure 1d, as well. The same JDE is observed, albeit with smaller magnitude. Qualitatively identical results were also observed for device G4, as reported in the SI.

We use the difference in the switching currents  to quantify the JDE. The dependence of Δ*I*_sw_ on magnetic field is presented in Figure 2b. To consider the asymmetry beyond the fluctuations due to stochastic switching, we performed a gentle smoothing procedure, as described in the SI. The experiment was repeated for different relative orientations of the magnetic field, as sketched in Figure 2a, to collect information about the angular dependence of the JDE. All measurements in Figure 2b show antisymmetric curves, i.e., . Furthermore, the curve for θ = −152° is flipped with respect to the others. This is consistent with the different orientations of the devices with respect to the field direction; i.e., the polarity of the Δ*I*_*sw*_ curves reflects the sign of the angle θ, which suggests that . We observe that Δ*I*_sw_ varies smoothly from a linear regime around zero field via a smooth rounded maximum at intermediate-field values to the high-field region, in which the effect is completely suppressed. The general trend is consistent with previous experiments, in which however a more rapid quenching was observed.^13^ To highlight the linear regime around zero field, we have added linear best fits at the origin of each curve (negative and positive branch fitted independently). A similar and consistent behavior was also observed for the retrapping current, as shown in the SI.

**Figure 2:**
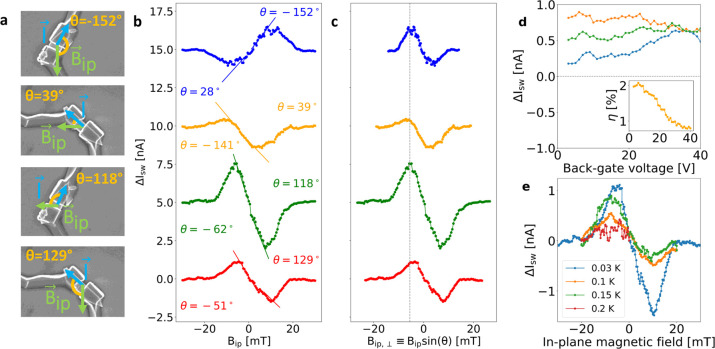
Behavior of the JDE with in-plane magnetic field, perpendicular component of the in-plane field, back-gate voltage, and temperature. (a) SEM images indicating the relative angle between *B*_ip_ and *I* (for positive field) for the measurements shown in panels b and c. For negative field, the angle is offset by 180°. (b) Asymmetry versus in-plane field for different orientations of the devices. Here, the blue and green curves correspond to device G4, while the yellow and red curves correspond to G5. Linear fits for small values of the field are also shown (negative and positive branch fitted independently). (c) Asymmetry versus the component of the magnetic field perpendicular to the current flow. The maximum of Δ*I*_sw_ is observed for *B*_ip,⊥_ = −6 ± 1 mT for each curve (indicated by the dashed line). The amplitude of the effect is maximum when θ is close to ±90°, i.e., when the in–plane magnetic field is perpendicular to the current vector, as explained in the main text. Note that the polarity of the curve at θ = −152° in panel c is reversed with respect to panel b due to the sign of sin(θ). In panels b and c, the curves are offset by 5 nA each for clarity. (d) Asymmetry versus back-gate voltage, for three different values of the applied in-plane magnetic field: blue, *B*_ip_ = −6 mT; orange, *B*_ip_ = −8 mT; green, *B*_ip_ = −10 mT. The inset shows the diode rectification coefficient η as a function of back-gate voltage, for *B*_ip_ = −8 mT. (e) Temperature dependence of the asymmetry. For panels d and e, Device G5.

To disentangle the contributions of the parallel (*B*_ip,∥_) and perpendicular (*B*_ip,⊥_) components of the field, computed with respect to the direction of current flow, we mapped in Figure 2c the measured data on the effective *B*_ip,⊥_. Note that, in the case of θ < 0, the change in polarity is due to the sign of sin(θ). In all data sets, the maximum asymmetry is observed for *B*_ip,⊥_ = −6 ± 1 mT, while its magnitude depends on the specific orientation. Thus, the main contribution to the effect is given by the perpendicular component of the field, consistent with .

Next, we study the dependence of the JDE on back-gate voltage. By setting the value of the field near the maximal Δ*I*_sw_ value of Device G5, we performed back-gate sweeps to the pinch-off of the devices. As shown in Figure 2d, the asymmetry is nearly constant in the explored range, which implies that the applied electrical field is not strong enough to significantly modulate spin–orbit coupling, consistently with results in similar systems.^13^ On the other hand, the back gate modulates the carrier concentration very efficiently in these devices,^47^ resulting in a reduction of the switching current from ∼50 nA to pinch-off in the same range,^48^ see also the SI. Thus, Δ*I*_sw_ appears to be robust against variations in carrier concentration and therefore seems to be governed by a mechanism other than *I*_sw_ itself. On the other hand, the relative strength of Δ*I*_sw_, or the diode rectification coefficient , which is the proper figure of merit to quantify the rectification effect, *increases* with decreasing gate voltage, as shown in the inset to Figure 2d.

Finally, in Figure 2e we show the influence of temperature. The amplitude of the asymmetry is rapidly reduced with increasing temperature and strongly suppressed already for *T* = 200 mK. We note that the acquisition at *T* = 150 mK is less antisymmetric, which we attribute to stochastic noise. Remarkably, while the diode effect disappears, the switching current at *T* = 200 mK is only reduced by about 20% with respect to its value at base temperature. On the other hand, the magnetic field value at which the maximum value of Δ*I*_sw_ is observed does not depend on temperature.

The same measurement as in Figure 1c is repeated in an out-of-plane magnetic field (no in-plane component), as shown in the inset to Figure 3a. In this case, no asymmetry is observed, consistent with previous results for similar systems.^43,48,51,52^ Finally, we add that all measurements performed at *B* = 0 resulted in asymmetry values equal to zero within the noise level.

**Figure 3:**
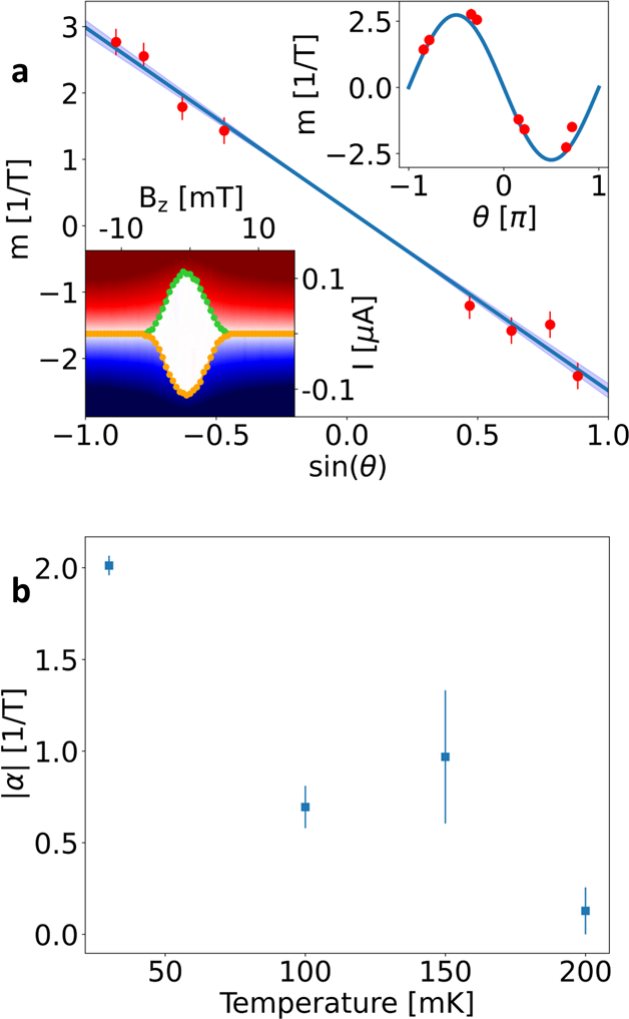
(a) Proportionality factor *m* (between rectification coefficient η and in-plane magnetic field *B*_ip_, i.e., *m* = η/*B*_ip_, see main text), plotted versus the sine of the angle θ between *B*_ip_ and the current flow direction. The blue line represents the best linear fit to the data, from which the value of α is extracted. The shaded region indicates the confidence interval. Upper inset: The same data plotted versus θ. The blue curve is a sine function, showing that the data points nicely follow a sin(θ) behavior. Lower inset: Measurement of the switching current with respect to a magnetic field perpendicular to the junction plane in a similar device, with the same material and geometric parameters of G4 and G5. The figure shows the voltage drop across the junction versus applied current bias *I* and out-of-plane magnetic field *B*_*z*_. The green (orange) dots indicate the positive (negative) switching currents, as defined in the main text. The width of the interference pattern is 4 times smaller than what we have obtained with an in-plane field. No asymmetry is observed in this configuration. (b) Proportionality factor |α| plotted versus *T*, for θ = 129° (Device G5).

In ref (53), it has been shown that *either* the presence of an in-plane field parallel to the current direction and a Dresselhaus SOC, *or* an in-plane field perpendicular to the charge flow and a Rashba SOC is a sufficient condition for this effect to emerge. Thus, the determination of which parameter drives the JDE provides valuable information about the key acting mechanisms in the junction. Here, we have measured the JDE for different angles θ, i.e., for different relative strength of the two in-plane components. We have shown in Figure 2c that the magnitude of the effect increases with the sine of the relative angle, i.e., with the perpendicular component of the in-plane field. Since the effect of this component is mediated by the Rashba coefficient, we can state that here a key role is played by the Rashba SO interaction. On the other hand, as shown in the SI, we have observed no clear trend with the parallel component, indicating that the Dresselhaus term is of little relevance in this system, consistent with previous results reported for InAs-based JJs.^15^

Our experimental evidence presented in Figure 2c shows that the behavior of Δ*I*_sw_ is antisymmetric with respect to *B*_ip,⊥_, and its maximum value is reached for *B*_ip,⊥_ = −6 mT, independent of back-gate voltage, temperature, or the relative angle θ. On the other hand, the magnitude of the effect does depend on the relative angle. The analysis in Figure 2b shows that the asymmetry depends linearly on the in-plane magnetic field near *B*_*ip*_ = 0, consistent with previous experimental results^11^ and theoretical predictions.^29^

To investigate the physics of this system, we consider models for the JDE in short ballistic junctions.^54,55^ These models are based on the idea of finite momentum Cooper pairs via the Zeeman effect on spin–helical electrons,^29,42,56^ akin to a so-called Doppler shift. In the InSb, the magnetic field introduces a Zeeman splitting term and, due to the strong SOC of the material, determines a spatially varying order parameter in the junction.^56^ Consequently, the Cooper pairs acquire a finite momentum *q* in the direction perpendicular to the magnetic field and the SO vector. This breaks the equivalence between the two propagation directions *I*^+^ and *I*^–^ of the current. We remark that here the spatial modulation occurs in the normal region of the junction and not in the superconducting leads.

If *E*_so_ is much larger than the Zeeman energy *E*_*z*_ = *g*_ip_^*^μ_B_*B*_ip_ ≪ *E*_so_, energy bands of opposite spin are split, and a finite Cooper pair momentum is expected.^56^ This condition is fulfilled here, since *E*_*z*_ = 15 μeV at 10 mT and thus much smaller than *E*_so_ = 200 μeV. Then, *qv*_F_ = *E*_*z*_, with *v*_F_ the Fermi velocity, and thus *q* ∝ *B*.^54,56^ The difference between the magnitudes of the critical currents in opposite directions  can then be calculated for small *B*, zero temperature, and one conductive channel as^54,55^
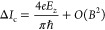
1Up to first order in the magnetic field, we also obtain
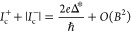
2with *e* being the electron charge. This finally allows the estimation of the diode rectification coefficient η in the linear-in-field regime:
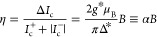
3Using the parameters for InSb (*g*_ip_^*^ = 25 and Δ* = 108 μeV, see the SI), we obtain α = 8.5 T^–1^ or equivalently a characteristic field *B*_0_ = 1/α = 118 mT.

To compare this result with the experiment, for each curve shown in Figure 2b, we extract the slope *m* of the linear fit of Δ*I*_sw_ near *B*_ip_ = 0 (negative and positive branches fitted independently). In Figure 3a, the values of *m* are plotted versus the sine of the relative angle θ, normalized to the sum of the two switching currents at zero field (red dots). The blue line is the result of a linear fit, from which the linear coefficient α = −2.9 ± 0.2 T^–1^ is extracted (corresponding to *B*_0_ = 345 mT), while the value of the intercept is negligible (β = 0.03 T^–1^). The relation *m* ∝ sin(θ) indicates that the rectification effect increases with the perpendicular component of the in-plane field *B*_ip,⊥_ = *B*_ip_ sin(θ).

By considering the behavior of Δ*I*_c_ at a finite field,^54,55^ we obtain that the maximum is reached for  (see the SI). Here it would thus be expected to be at *B* = 58 mT, which is higher than experimentally observed. We attribute the discrepancy to the presence of the parallel component of *B*_ip_, which is expected to suppress the supercurrent flow at higher fields. Moreover, the model does not consider other effects due to the finite size of the junctions, which could be relevant in our system, as well.

The temperature dependence of the asymmetry curves, shown in Figure 2e, deserves attention. In fact, whereas the switching current hardly varies in the temperature range 30–200 mK, the JDE undergoes a nearly complete suppression. Correspondingly, the rectification coefficient |α| is strongly reduced with increasing temperature (see Figure 3b). The differing behavior between these two quantities originates from the fact that the JDE is strongly dependent on the presence of higher harmonics in the CPR of ballistic SNS junctions.^13^ Indeed, in the case of a purely sinusoidal dependence, the anomalous phase shift does not induce any difference between *I*_sw_^+^ and *I*_sw_^–^, which correspond to the maximum and minimum of the CPR, respectively. Higher harmonics decay faster with increasing temperature, so that in the high-temperature limit, the only relevant harmonic is the lowest one; i.e, the CPR is a simple sine function. Thus, the JDE is strongly suppressed in temperature, due to the much stronger dependence of the higher harmonics with respect to the fundamental one.

In conclusion, we have demonstrated that a single planar JJ made from an InSb nanoflag can be driven into the nonreciprocal transport regime by an in-plane magnetic field applied perpendicularly to the direction of the current flow. Moreover, the extent of the rectification depends on the specific combination of the two in-plane field components. Based on symmetry arguments, we have determined that a key role is played by the Rashba SOC. Furthermore, we have elucidated the dependence of the effect on other parameters and, specifically, that increasing temperature drastically quenches supercurrent rectification. This is consistent with the absence of higher harmonics in the CPR expected at elevated temperature.

Thus, high-quality InSb nanoflags are optimal candidates to realize low-dissipation supercurrent rectifiers and to explore the physics of nonreciprocal superconductivity. Further progress in this field will be promoted by the development of microscopic theories which link the rectification quantitatively to the spin–orbit coupling strength. Then, we expect the SDE to become a useful addition to the toolbox of hybrid superconducting electronics.

## Supporting Information Available

Additional details and results including sample characterization, fitting procedure, experimental data, raw data (PDF)
