# Supplementary material for: Josephson Diode Effect in High-Mobility InSb Nanoflags
Source: Nano Lett. 2022 Oct 26;22(21):8502–8. doi: 10.1021/acs.nanolett.2c02899 (PMC9650771; doi:10.1021/acs.nanolett.2c02899)
Supplement: Supplementary file 1 — nl2c02899_si_001.pdf [file nl2c02899_si_001.pdf]

Supporting Information
Josephson Diode Effect in High Mobility InSb Nanoflags

Bianca Turini,† Sedighe Salimian,† Matteo Carrega,‡ Andrea Iorio,† Elia Strambini,† Francesco Giazotto,† Valentina Zannier,† Lucia Sorba,† and Stefan Heun*,†

†*NEST, Istituto Nanoscienze-CNR and Scuola Normale Superiore, 56127 Pisa, Italy*
‡*CNR-SPIN, 16146 Genova, Italy*

E-mail: stefan.heun@nano.cnr.it

Sample characterization

The InSb nanoflags used in this work and the Nb/Ti–InSb JJs were extensively characterized in previous publications.[1,2] The InSb nanoflags are defect–free zinc–blende structures with high mobility (up to $\mu_e \sim 29500$ cm^{2}V^{-1}s^{-1}) and a large mean free path ($\lambda_e = 500$ nm) at $T = 4.2$ K.[1] We note that the extracted Fermi wavelength ($\lambda_F = 2\pi/k_F = \sqrt{2\pi/n} \sim 30$ nm for carrier concentration $n = 8.5 \times 10^{11}$ cm^{-2} [1]) is of the same order of magnitude as the thickness of the nanoflags ($t \approx 100$ nm):[1,2] by evaluating the number of the active transport modes (≈ 40)[2] and the degeneracy of the vertical subbands, we find that these devices are well placed in the quantum limit with a clear 2D character.

For device fabrication, the nanoflags are placed on a p–doped Si/SiO$_2$ substrate, which acts as a global back–gate. The nanoflags are contacted by 10/150 nm of Ti/Nb, which

defines the superconducting leads, leaving the central region of the nanoflag uncovered. The dimensions of the resulting planar Josephson junctions, length $L = 200$ nm and width $W = 700$ nm, are such that the devices work in the ballistic regime. More details on device fabrication can be found in the supplemental material of Ref. 2. Figure S1a and Figure S1b show the two devices discussed in this manuscript, named G4 and G5, respectively. The two devices, resulting from the same fabrication process, are characterized by the same material and geometric parameters. The superconducting coherence length can be determined as $\xi_s = \hbar v_F/\Delta$,[3–6] where Δ is the gap in the superconductor and v_F the Fermi velocity in the semiconductor. By inserting the value of the Nb gap ($\Delta = 1.28$ meV[2]) and the value of the Fermi velocity of the InSb nanoflags ($v_F = \hbar k_F/m^* = 1.5 \times 10^6$ m/s[2]), we obtain a coherence length much larger than the length of the uncovered region ($\xi_s \approx 750$ nm $> L$). Thus, the devices operate in the short junction regime.

Transport measurements were performed in an Oxford Triton 200 dilution refrigerator with a base temperature of 30 mK. The measurement setup is sketched in Figure S1c. We study the low-temperature magneto-transport of the devices in the presence of an in-plane magnetic field. A relative angle θ ($-180° \leq \theta \leq 180°$) can be set between the orientation of the in-plane magnetic field and the direction of current flow, $\vec{B}_{ip}$ and $\vec{I}$, respectively. The sign of θ is given by the direction of the $\vec{B}_{ip} \times \vec{I}$ vector. With this definition, $\theta = 0°$ for $\vec{B}_{ip} \parallel \vec{I}$ and $\theta = 90°$ for $\vec{B}_{ip} \perp \vec{I}$.

Figure S1d shows a characteristic $V - I$ curve of device G4 measured at $T = 30$ mK and $V_{bg} = 40$ V. We can clearly distinguish the switching (I_{sw}) and the retrapping (I_{rt}) currents.[7] Figure S1e shows that the extent of the superconducting region decreases monotonically with increasing temperature. The data in the 2D plot are collected by performing a sweep from negative to positive bias, so that the upper plane shows the switching current, while the lower one presents the retrapping value. We also measured the opposite sweep direction (from positive to negative bias, see Figure S2). For both sweep directions, we extracted the values of I_{sw} and I_{rt}. They are shown as dots in Figure S1e, overlaid on the 2D plot. The

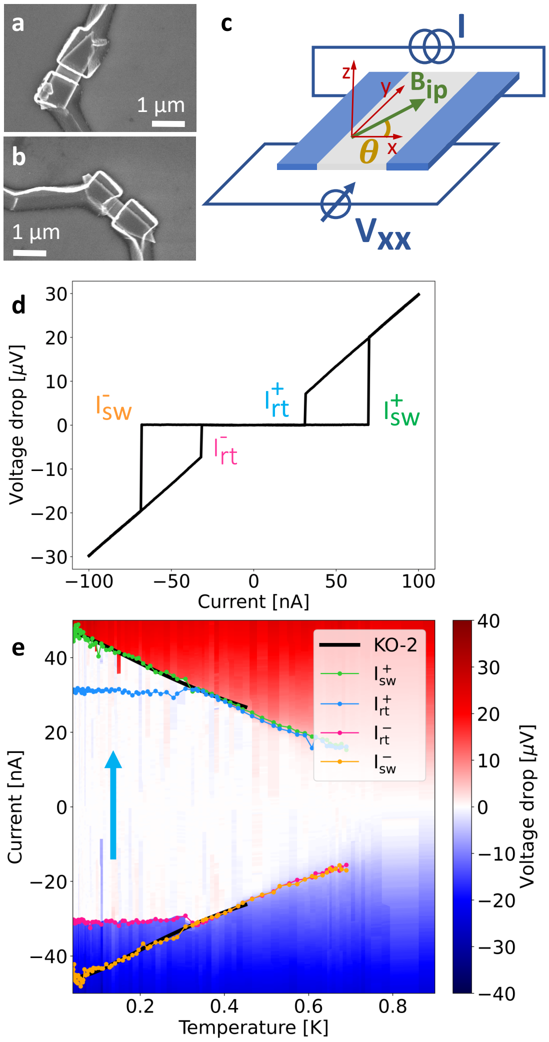

Figure S1: Device characterization. (a,b) SEM images of the two devices, G4 and G5. (c) Sketch of the measurement schematics. Also the angle θ between the orientation of the in-plane magnetic field B_{ip} and the direction of current flow I is indicated. (d) $V-I$ characteristics at $T = 30$ mK. The difference between switching and retrapping current, defined in the plot, is clearly visible. (e) Temperature dependence of the $V-I$ characteristics. The individual spectra were measured sweeping from negative to positive bias values, in the direction indicated by the arrow. Thus, the 2D plot shows the switching trace for positive values of the current and the retrapping trace for the negative ones. The extracted values of $I^+_{sw}, I^+_{rt}, I^-_{sw}, I^-_{rt}$ for each temperature and both sweep directions are shown in green, blue, pink and orange, respectively. The black lines show a fit of the switching currents to the KO-2 model (see text). For (d,e): device G4, $B = 0$ mT, and $V_{bg} = 40$ V.

values of I_{sw} and I_{rt} differ for temperatures lower than ~ 300 mK, consistent with previous measurements.[2] This hysteretic behavior is typical of SNS weak-links[8,9] and is commonly understood as Joule heating of the N region in the dissipative regime.[10–13] In all the following arguments, the switching and retrapping currents are considered separately.

Figure S1e shows the $V - I$ characteristics dependence on temperature for one sweep direction only (from negative to positive current values). For completeness, we report the data acquired in the other direction in Figure S2. In this case, we observe the *retrapping* current values in the positive branch and the *switching* current values for the negative one.

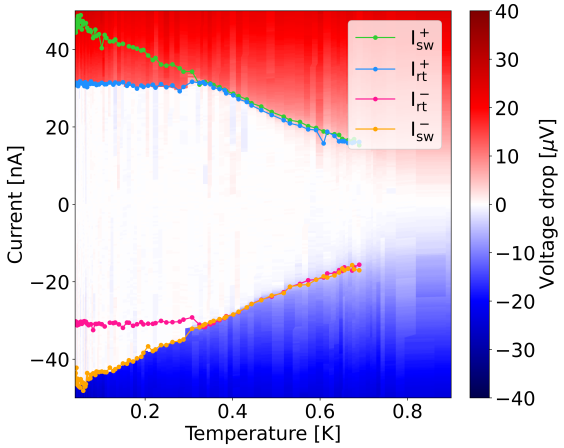

Figure S2: Temperature dependence of the $V-I$ characteristics. The individual spectra were measured sweeping from positive to negative bias. Thus, the 2D plot shows the retrapping trace for positive values of the current and the switching trace for the negative ones. The extracted values of I^+_{sw}, I^+_{rt}, I^-_{rt}, I^-_{sw} for each temperature and both sweep directions are shown in green, blue, pink and orange, respectively. Device G4, $B = 0$ mT, and $V_{bg} = 40$ V.

Fitting procedure for the dependence $I_{sw} - T$

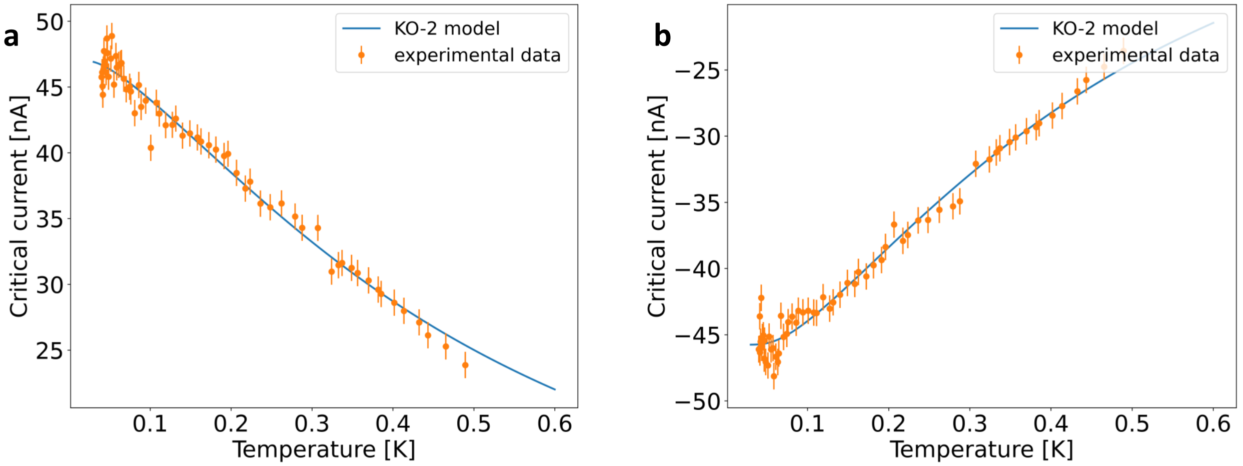

Figure S3: Temperature dependence of (a) the positive and (b) the negative switching current. The experimental data are shown as orange dots, together with the measurement error. The blue line is the best fit curve described by the KO-2 model.

The value of the switching current for each temperature is extracted from the 2D maps in Figure S1e and Figure S2. The temperature dependence of the switching current is well described by the Kulik–Omelyanchuk model in the clean limit (KO–2).[14–16] In this case, the current-phase relation at $B = 0$ is described by the following equation:

$$I(\varphi, \tau, T) = I_0 \frac{\sin \varphi}{\sqrt{1 - \tau \sin^2 \varphi/2}} \tanh \left(\frac{\Delta^*}{2k_B T} \sqrt{1 - \tau \sin^2 \varphi/2} \right), \tag{1}$$

where φ is the superconducting phase difference across the junction, τ the transmission coefficient averaged over the transport modes, T the temperature, and I_0 is the critical current for $T = 0$. The induced gap Δ^* is taken as a constant in the range of temperatures under study, thus the temperature range considered for the fit is 0.03 K $< T <$ 0.5 K. The value of the positive (negative) critical current is defined as the maximum (minimum) of this function with respect to the phase

$$I_c^+ = \max_{\varphi} I(\varphi, \tau, T) \qquad I_c^- = \min_{\varphi} I(\varphi, \tau, T). \tag{2}$$

The absolute error for the switching current is $\sigma = 1$ nA. The best fit function for the positive and negative branches are reported, together with the experimental data, in Figure S3a and Figure S3b, respectively.

The optimal parameters are consistent for the two data sets. For the positive branch, we obtain:

$$I_0 = (48.1 \pm 0.7) \text{ nA}$$
$$\Delta^* = (111 \pm 3) \quad \mu\text{eV}$$
$$\tau = (0.99 \pm 0.01)$$

Consistent values are obtained for the negative branch:

$$I_0 = (-49 \pm 1) \text{ nA}$$
$$\Delta^* = (104 \pm 4) \quad \mu\text{eV}$$
$$\tau = (0.99 \pm 0.01).$$

The best fits are reported, together with the experimental data, in Figure S3. The good agreement with the KO-2 model indicates that the junctions are working in the ballistic regime, as expected from the value of $\lambda_e > L$. This confirms that the devices are in the ballistic short–junction regime, which leads to a skewed CPR including higher harmonics, crucial for observing the JDE.[17] The extracted value for the induced gap is $\Delta^* = 108 \pm 4$ μeV, consistent with the values found in literature.[2] The resulting transmission probability $\tau \sim 0.99$ confirms the high quality of the interfaces in the devices, as previously reported.[2]

Experimental data from device G4

Device G4 shows in average higher values of switching current at low fields, but it is also more noisy. Figure S4a shows the 2D map of voltage drop *versus* in-plane magnetic field (x axis) and current (y axis), for $\theta = -152°$. The skewness of the pattern is reversed with respect to what shown in Figure 1 of the main text, consistently with the difference of angle between the two devices (see main text). In this case, the asymmetry in the retrapping current is not visible with our experimental resolution.

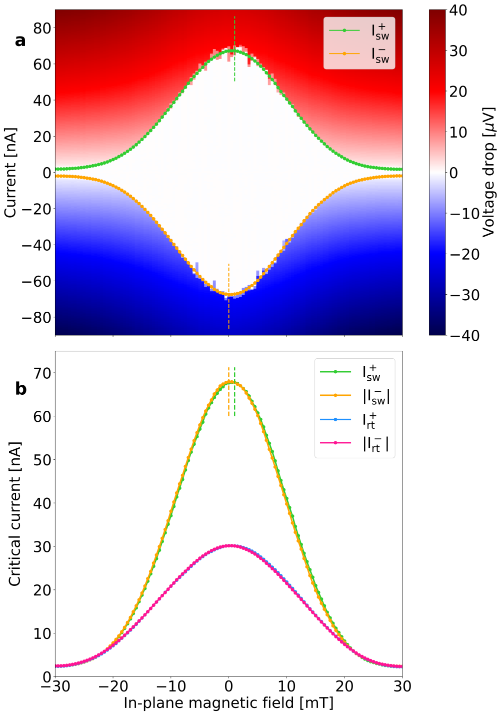

Figure S4: Switching current dependence on in-plane magnetic field. (a) Voltage drop across the junction versus in-plane magnetic field. The superconducting phase, defined by dissipation–less charge transport, corresponds to the white region. The green (orange) dots indicate the positive (negative) switching current, as defined in the main text. (b) The switching current shows a clear asymmetry between the positive and negative branches, shown in green and orange, respectively. The negative branch is higher for positive values of the magnetic field, and the relation is reversed for negative field. The blue and pink lines correspond to the positive and negative retrapping current. Device G4, $T = 30$ mK, and $V_{bg} = 40$ V.

Raw data for ΔI_{sw}

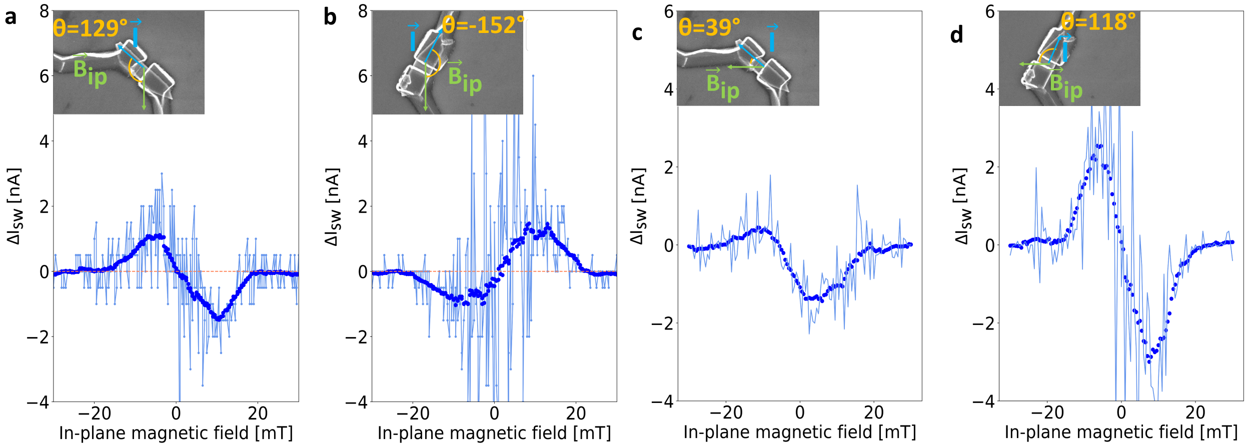

Figure S5: Asymmetry in the switching current with respect to the in–plane magnetic field, for four different relative orientations between the current flow and the magnetic field direction, as indicated in the insets. (a) Asymmetry for Device G5, which is placed at an angle of $\theta = 129°$ with respect to the field direction. (b) Device G4, $\theta = -152°$. (c) Device G5, $\theta = 39°$. (d) Device G4, $\theta = 118°$. For all measurements, $T = 30$ mK and $V_{bg} = 40$ V.

In Figure S5 we show raw data for all the measured orientations, together with the curves resulting from the smoothing procedure, as explained in the following.

The InSb nanoflags under study exhibit stochastic switching, i.e. the value of the switching current is a stochastic quantity, especially at low temperature. For this reason, the dependence of the switching current is masked with noise. This phenomenon is due to quantum fluctuations and of great interest in the study of phase dynamics.[18]

To distinguish the general trend from fluctuations, we have performed a smoothing procedure of the data with the Savitzky-Golay (SG) filter.[19] The SG algorithm is defined by two parameters: the width (W) of the window of data to be considered in the analysis and the order (n) of the polynomial expression used in the least-square fit. In Figure 2 of the main text and in Figure S4, the chosen parameters are $W = 31$, $n = 2$.

In Figure S5a,b, the data from the first acquisition set are reported. The angle θ (see main text) was set to $\theta = -152°$ for G4 and $\theta = 129°$ for G5. The light blue lines stand for the raw data, while the blue dots represent the result of the smoothing procedure. Here, we

have used $W = 31$, $n = 1$ for smoothing.

In Figure S5c,d, the angle θ was set to $\theta = 118°$ for G4 and $\theta = 39°$ for G5. In this case, 17 curves were acquired for each value of magnetic field, resulting in 17 values of asymmetry, which are averaged before performing the smoothing procedure. The averaged values are represented by the light blue line, while the smoothed data are shown as blue dots. The smoothing parameters are $W = 15$, $n = 1$.

JDE in the retrapping current

The switching and the retrapping current are clearly distinguishable in these systems, up to $T \sim 300$ mK. We have observed the JDE also in the retrapping data, even if weaker. Considering that the difference between the two is related to the higher effective temperature of the electron gas in the retrapping branch, we can use the same arguments as in Section (the temperature dependence of the JDE) to explain this reduction.

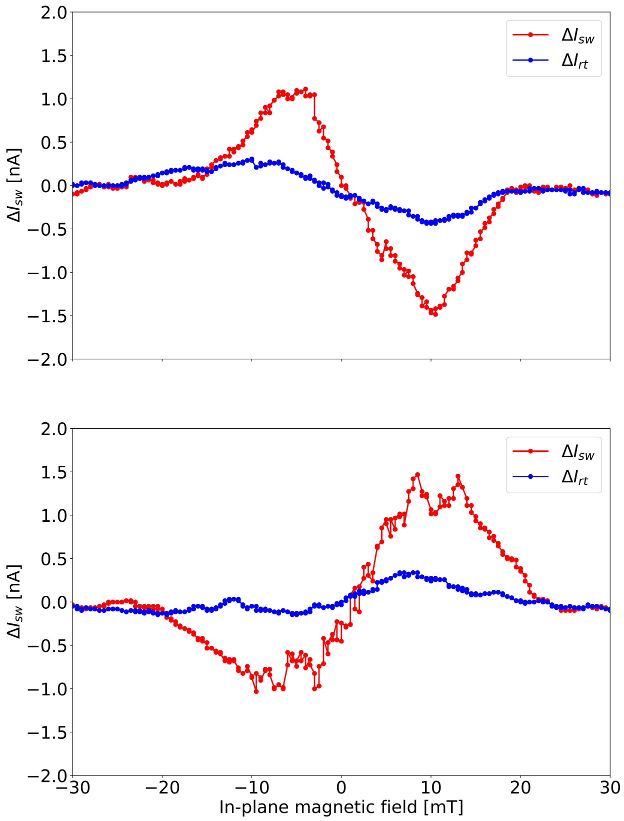

Figure S6: Asymmetry in the switching and retrapping current with respect to the in–plane magnetic field, represented by the red and blue dots, respectively. The effect is strongly reduced in the latter, while the polarity and the shape of the curves are maintained.

Parallel component of the in-plane magnetic field

To disentangle the contributions of the parallel ($B_{ip,\parallel}$) and perpendicular ($B_{ip,\perp}$) components of the in-plane magnetic field, computed with respect to the direction of current flow, we mapped the measured data on the effective $B_{ip,\parallel} = B_{ip}\cos(\theta)$. In this case, there is a clear mismatch in the shape of the asymmetry for different angles. More importantly, while the polarity in the $B_{ip,\perp}$ dependence is the same for each θ (see Figure 2c of the main text), this is not observed in ΔI_{sw} vs. $B_{ip,\parallel}$. This evidence is consistent with the interpretation of a dominant Rashba-type SOI (see main text).

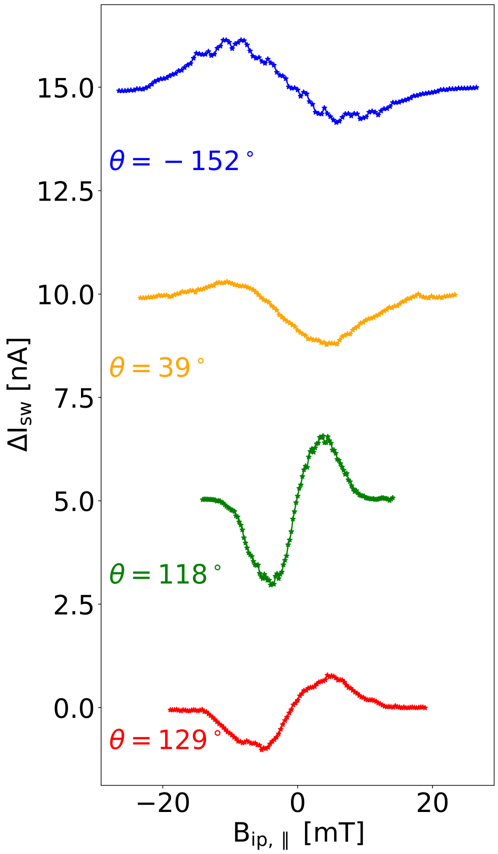

Figure S7: Behavior of JDE with parallel component of the in-plane magnetic field. Asymmetry versus the component of the magnetic field parallel to the current flow. No clear trend of visible. The curves are shifted by 5 nA each for clarity.

Back-gate dependence of the JDE

As previously shown,[2] these devices act as Jo-FETs, i.e., the switching current is tunable with gate voltage (V_{bg}). In Figure S8a, measured at $B = 0$, the 2D map shows how the extent of the supercurrent changes with V_{bg}. Data in Figure S8b–d are sampled in presence of a magnetic field of $B_{ip} = -6$ mT, $B_{ip} = -8$ mT, and $B_{ip} = -10$ mT, respectively. The resulting asymmetry is shown in Figure 2d of the main text. Despite the strong variation that the field effect induces on I_{sw}, which is over 50 nA in the range considered, there is no visible effect on ΔI_{sw} (see main text).

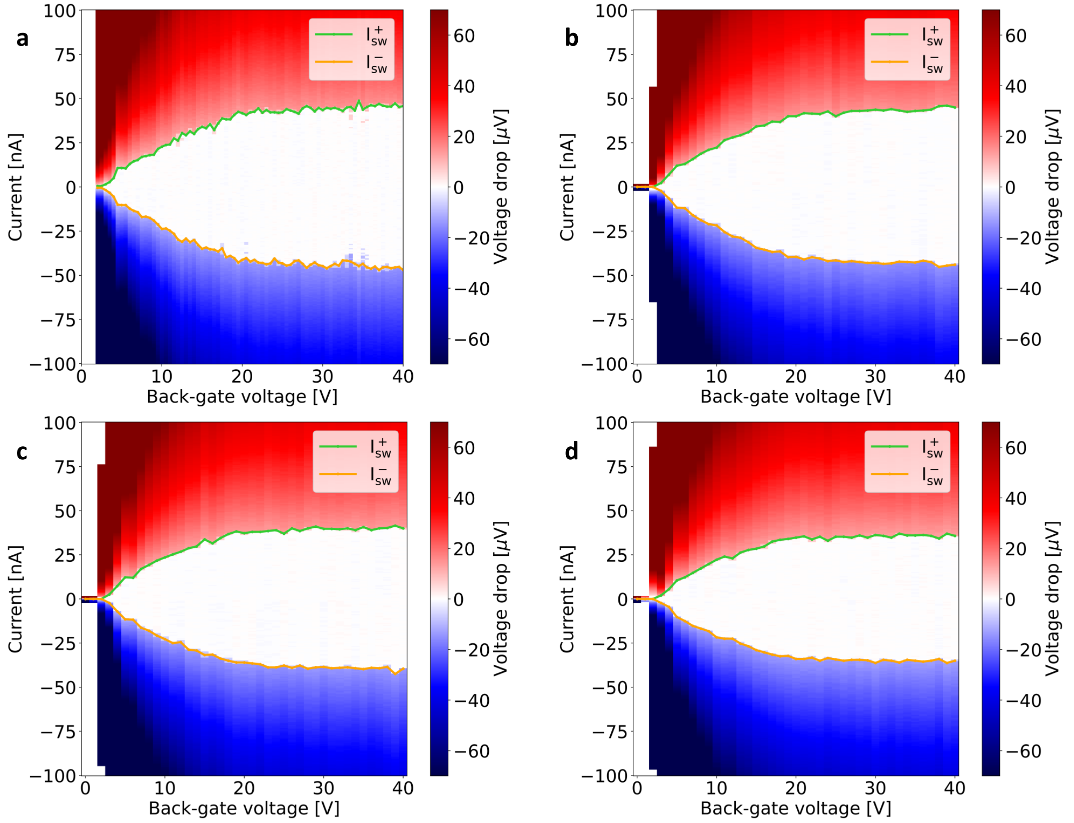

Figure S8: Electrostatic control of the switching current. The color map represents the voltage drop as a function of back gate voltage and current. The in–plane magnetic field was fixed to different values for each acquisition: (a) $B_{ip} = 0$, (b) $B_{ip} = -6$ mT, (c) $B_{ip} = -8$ mT, and (d) $B_{ip} = -10$ mT. The positive (negative) switching current I_{sw}^{+} (I_{sw}^{-}) is shown with green (orange) dots. Device G5, $T = 30$ mK.

Temperature dependence of the JDE

As explained in the main text, the JDE is strongly reduced with increasing temperature. Figure S9 shows the interference patterns for $T = 30$ mK, $T = 100$ mK, $T = 150$ mK, and $T = 200$ mK. The last acquisition was not completed since the data already demonstrated that the effect had faded completely. It is clear from the 2D maps that the value of the switching current is very little reduced for such small changes of temperature, consistent with Figure S1e.

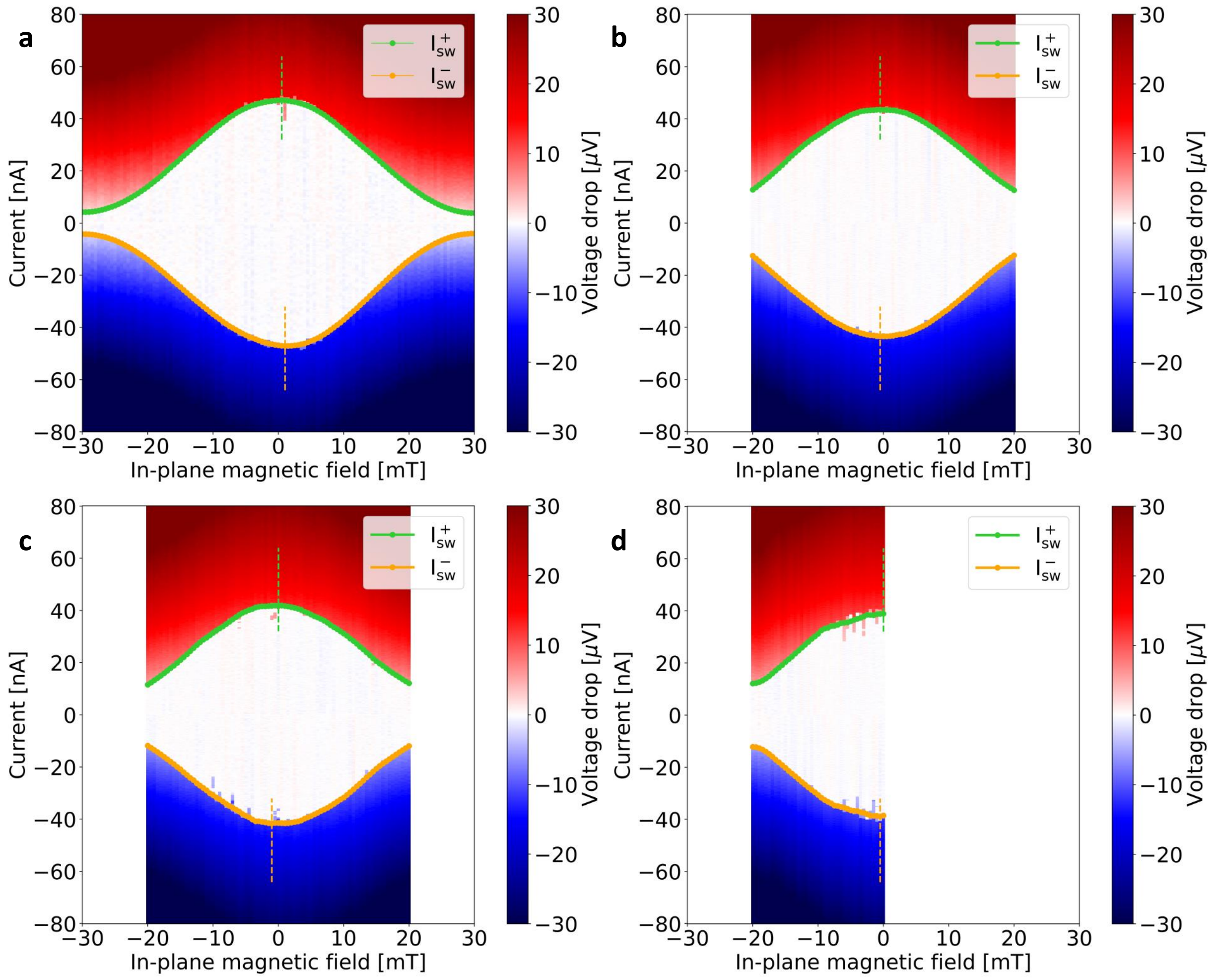

Figure S9: Interference patterns measured at different temperatures. The temperature was fixed to 30, 100, 150, 200 mK for the data in a,b,c,d, respectively. The positive (negative) switching current I^+_{sw} (I^-_{sw}) is shown with green (orange) dots.

Universal maximum in ΔI_{sw}

We find the maximum of the asymmetry ΔI_{sw} with respect to $B_{ip,\perp}$, by making use of the expression

$$\Delta I_c = \mathrm{sgn}(q)\left(\frac{4e|q|v_F}{\pi\hbar} - \frac{e\Delta^*}{\hbar}\left[1 - \sqrt{1 - \left(\frac{qv_F}{\Delta^*}\right)^2}\right]\right) \tag{3}$$

as reported in.[20] The maximum is reached for

$$q_0 v_F = \sqrt{\frac{16}{\pi^2 + 16}}\ \Delta^* \approx 0.78\,\Delta^*. \tag{4}$$

To compute the value of $B_{max} = 58$ mT, we use $\Delta^* = 108\ \mu$eV, $g^*_{ip} = 25$.[21,22]

References

(1) Verma, I.; Salimian, S.; Zannier, V.; Heun, S.; Rossi, F.; Ercolani, D.; Beltram, F.; Sorba, L. High-Mobility Free-Standing InSb Nanoflags Grown on InP Nanowire Stems for Quantum Devices. *ACS Appl. Nano Mater.* **2021**, *4*, 5825–5833.

(2) Salimian, S.; Carrega, M.; Verma, I.; Zannier, V.; Nowak, M. P.; Beltram, F.; Sorba, L.; Heun, S. Gate-controlled supercurrent in ballistic InSb nanoflag Josephson junctions. *Appl. Phys. Lett.* **2021**, *119*, 214004.

(3) Lee, G.-H.; Lee, H.-J. Proximity coupling in superconductor-graphene heterostructures. *Rep. Prog. Phys.* **2018**, *81*, 056502.

(4) de Vries, F. K.; Sol, M. L.; Gazibegovic, S.; op het Veld, R. L. M.; Balk, S. C.; Car, D.; Bakkers, E. P. A. M.; Kouwenhoven, L. P.; Shen, J. Crossed Andreev reflection in InSb flake Josephson junctions. *Phys. Rev. Research* **2019**, *1*, 032031.

(5) Zhi, J.; Kang, N.; Li, S.; Fan, D.; Su, F.; Pan, D.; Zhao, S.; Zhao, J.; Xu, H. Supercur-

rent and Multiple Andreev Reflections in InSb Nanosheet SNS Junctions. *Phys. Status Solidi B* **2019**, *256*, 1800538.

(6) Banszerus, L.; Libisch, F.; Ceruti, A.; Blien, S.; Watanabe, K.; Taniguchi, T.; Hüttel, A. K.; Beschoten, B.; Hassler, F.; Stampfer, C. Minigap and Andreev bound states in ballistic graphene. arXiv:2011.11471 [cond-mat.mes-hall], 2021.

(7) We note that the data of Ref. 2, for which this hysteretic behavior was not observed, were obtained at $T = 250$ mK, i.e., at a higher temperature than employed here.

(8) Tinkham, M. *Introduction to superconductivity*; McGraw-Hill, 1996.

(9) Guiducci, S.; Carrega, M.; Biasiol, G.; Sorba, L.; Beltram, F.; Heun, S. Toward Quantum Hall Effect in a Josephson Junction. *Phys. Status Solidi RRL* **2019**, *13*, 1800222.

(10) Schäpers, T. *Superconductor / semiconductor junctions*; Springer, 2001.

(11) Courtois, H.; Meschke, M.; Peltonen, J. T.; Pekola, J. P. Origin of Hysteresis in a Proximity Josephson Junction. *Phys. Rev. Lett.* **2008**, *101*, 067002.

(12) Fornieri, A. Josephson effect in ballistic semiconductor nanostructures. M.Sc. thesis, University of Pisa, 2013.

(13) Guiducci, S.; Carrega, M.; Taddei, F.; Biasiol, G.; Courtois, H.; Beltram, F.; Heun, S. Full electrostatic control of quantum interference in an extended trenched Josephson junction. *Phys. Rev. B* **2019**, *99*, 235419.

(14) Kulik, I. O.; Omel'yanchuk, A. N. Properties of superconducting microbridges in the pure limit. *Sov. J. Low Temp. Phys.* **1977**, *3*, 459–462.

(15) Golubov, A. A.; Kupriyanov, M. Y.; Il'ichev, E. The current-phase relation in Josephson junctions. *Rev. Mod. Phys.* **2004**, *76*, 411–469.

(16) Lee, G.-H.; Kim, S.; Jhi, S.-H.; Lee, H.-J. Ultimately short ballistic vertical graphene Josephson junctions. *Nature Communications* **2015**, *6*, 6181.

(17) Baumgartner, C.; Fuchs, L.; Costa, A.; Reinhardt, S.; Gronin, S.; Gardner, G. C.; Lindemann, T.; Manfra, M. J.; Faria Junior, P. E.; Kochan, D.; Fabian, J.; Paradiso, N.; Strunk, C. Supercurrent rectification and magnetochiral effects in symmetric Josephson junctions. *Nature Nanotechnology* **2022**, *17*, 39–44.

(18) Haxell, D. Z.; Cheah, E.; Křížek, F.; Schott, R.; Ritter, M. F.; Hinderling, M.; Belzig, W.; Bruder, C.; Wegscheider, W.; Riel, H.; Nichele, F. Large Quantum Fluctuations in Planar Josephson Junctions. arXiv:2204.05619 [cond-mat.mes-hall], 2022.

(19) Savitzky, A.; Golay, M. J. E. Smoothing and Differentiation of Data by Simplified Least Squares Procedures. *Analytical Chemistry* **1964**, *36*, 1627–1639.

(20) Davydova, M.; Prembabu, S.; Fu, L. Universal Josephson diode effect. *Science Advances* **2022**, *8*, eabo0309.

(21) Qu, F.; van Veen, J.; de Vries, F. K.; Beukman, A. J. A.; Wimmer, M.; Yi, W.; Kiselev, A. A.; Nguyen, B.-M.; Sokolich, M.; Manfra, M. J.; Nichele, F.; Marcus, C. M.; Kouwenhoven, L. P. Quantized Conductance and Large g-Factor Anisotropy in InSb Quantum Point Contacts. *Nano Lett.* **2016**, *16*, 7509–7513.

(22) de la Mata, M.; Leturcq, R.; Plissard, S. R.; Rolland, C.; Magén, C.; Arbiol, J.; Caroff, P. Twin-Induced InSb Nanosails: A Convenient High Mobility Quantum System. *Nano Lett.* **2016**, *16*, 825–833.
